# Simulation Modeling for Psychiatric Service Planning: Protocol for a Mixed-Methods Study

**DOI:** 10.2196/11119

**Published:** 2018-11-23

**Authors:** Katrina M Long, Graham N Meadows

**Affiliations:** 1 Southern Synergy Department of Psychiatry Monash University Dandenong Australia; 2 Melbourne School of Population and Global Health University of Melbourne Melbourne Australia; 3 Monash Health Melbourne Australia

**Keywords:** decision making, health care, health services administration, mental health, protocol, mixed-methods, modeling, simulation

## Abstract

**Background:**

Mental health service managers must take into account multiple factors when making decisions about the best way to deliver care to clients across increasingly larger service areas. This task is made more difficult by the lack of evidence and tools historically available to inform these decisions. In recent decades, the increasing availability of epidemiological and service use data for mental illness has solved the problem of evidence, but there still exists a challenge to make these data easily accessible and understandable for managers.

**Objective:**

This study aims to develop a simulation modeling tool to allow managers to explore various service configurations in virtual reality, enabling predictions to be made about the cost and quality of care.

**Methods:**

This is a longitudinal, mixed-methods case study, comprising overlapping intervention and evaluation phases. In partnership with senior managers of a mental health program, the researchers will develop a series of simulation models in Arena to address key strategic issues facing the service. Thematic and content analyses of semistructured interviews, meeting observations, and document analysis will be used to evaluate the process of model implementation and the outcomes for both researchers and managers. The study is being conducted in Australia.

**Results:**

Data collection has been ongoing since late 2013. To date, 3 prototype simulation models have been developed and presented to senior managers, and 18 evaluation interviews have been conducted. The project is expected to conclude in late 2018.

**Conclusions:**

Findings of this study have the potential to shape decision making in mental health service delivery, by providing key examples of how to integrate patient data using simulation modeling. In addition, the results will provide key insights into how researchers and consultants can effectively implement simulation modeling in real-world health care organizations.

**International Registered Report Identifier (IRRID):**

RR1-10.2196/11119

## Introduction

The health care sector is characterized by complexity, where balancing the demands of multiple stakeholders in geographically disparate areas makes the task of service-wide strategy planning extraordinarily difficult [1,2]. In mental health, this is exacerbated by the heterogeneity of illness severity, persistence, treatment response, and treatment need, as well as the multitude of entry points and patient pathways through the mental health system [3].

In the clinical space, this complex environment is managed through the use of evidence-based practice [4] and clinical simulations to provide staff with decision-making experience in a low-risk environment [5]. In health care management, mechanisms for evidence-based decision making are much less ubiquitous. Instead, managers have traditionally relied on personal knowledge and experience to make small incremental service changes within a quality improvement framework [6]. Unfortunately, the inherent risks of the “try it and see” approach make it unsuitable for the large-scale service reforms currently being called for in the Australian mental health sector [7]. Thankfully, ongoing improvements in technology and electronic patient records have created a fertile environment for the translation of decision support tools from other sectors, including that of simulation modeling.

Simulation models are simplified abstractions of real systems, often created on a computer. They allow users to predict future states by tracking changes in the system over time, with these changes determined by attributes assigned to individuals or entities (agent-based modeling), time-specific state transitions (Markov models), events (discrete event simulation), or system flows (system dynamics) [8]. Simulation modeling is claimed to improve the rationality of decision makers and therefore improve decision quality [9], by allowing problem boundaries and alternatives to be explored safely and inexpensively [10,11].

However, little direct evidence is provided to support these claims of improved decision making outcomes. This is due to a general lack of reporting on the implementation of simulation models, with multiple reviews of health care simulation highlighting this as a key problem facing the literature [12-18]. Indeed, a recent review of mental health care simulation found only 10 papers reporting basic details of model implementation [8]. While this lack of reporting may reflect publication bias, it more likely reflects the difficulty in implementation, including the time and financial costs associated with increasing model complexity to match the clinical complexity of the health care environment. However, it is this very complexity that calls for the use of simulation and the transparent reporting of implementation.

Hence, this paper aims to describe the protocol for the development and implementation evaluation of a simulation model depicting the real-world activities of an Australian public mental health service (MHS).

The primary aims of this study are (1) to develop a sophisticated health care management decision support tool and bring it into practical use by managers of MHS as they go about service reform and redevelopment and (2) to evaluate the effectiveness of this decision support tool in improving the process and outcome of strategic decision making by MHS managers.

## Methods

### Study Design

The intervention and evaluation follow an iterative, mixed-method design. The intervention and evaluation timelines are staggered, but intentionally overlap, to allow evaluation results to inform refinements to the intervention in the latter stages of the study. The intervention was designed and overseen by GNM, and the evaluation was designed and conducted by KML.

#### Intervention Design

**Figure 1:**
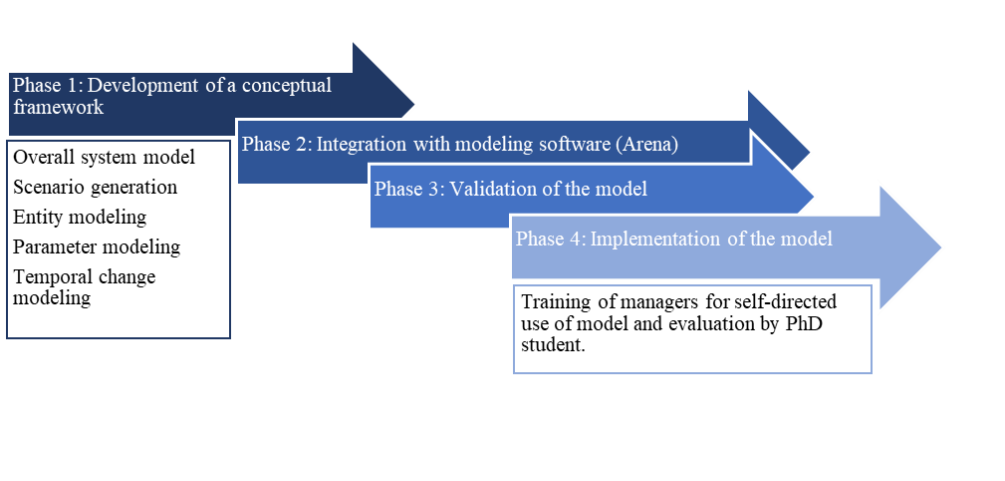
Intervention design.

The intervention has 4 major phases: (1) development of a conceptual framework for the simulation model; (2) integration with simulation software; (3) validation of the model; and (4) implementation of the model within the MHS (Figure 1). In the first phase, we will analyze the components and functionalities of a mental health system and develop the architecture of a generic framework for the simulation model so that it can be embedded into any commercially available simulation modeling tool. In the second phase, we will embed the framework into Arena simulation software (a widely used modeling tool). The third phase will involve extensive validation of the model using data from the MHS. In the final phase, the model will be implemented as a decision-making tool within the MHS. The tasks in the phases will occur in parallel with some overlap between phases to provide a mechanism for each component to benefit from the outcomes of the progressive development and evaluation.

#### Evaluation Design

The evaluation design is a longitudinal, mixed-method case study that parallels the intervention. The analysis focuses on 2 levels: outcome and process.

Outcome will be measured by changes in mental models, reflecting increased decision process agreement and increased similarity to the rational decision-making model [19]. In addition, the outcome will be measured by researcher and participant perceptions of the intervention success, behavioral change, and cognitive change, as extracted by the thematic analysis from exit interviews.

The process will be assessed by group changes in behavioral and linguistic patterns during the intervention workshops, reflecting increased similarity to the features of good group decision-making processes [20]. These observations will be triangulated against participants’ self-report of workshop success extracted by an evaluation questionnaire.

### Study Setting

The research was conducted with the cooperation of the senior leadership group (SLG) of a major public MHS in Australia. The MHS provides government-funded inpatient and community mental health services across the age spectrum, with different, but overlapping, catchment areas for Early in Life Mental Health Services (<25 years), adult, and aged (<65 years) services. There are 3 operational service groups, Early in Life Mental Health Services, community services, and bed-based services, and 3 primary hospital sites, which were added to the organizational chart in 2016. The MHS employs approximately 800 staff members who provide approximately 250,000 client contacts per year, at a total cost of Aus $125 million, 8.0% of the health provider’s operating expenditure.

Strategic decision making for the MHS lies with the SLG. Members of the group attend monthly meetings as representatives of their clinical specialty (psychiatrists, psychologists, allied health, and nurses), operational units, administrative units (finance and human resources), and allied research/university groups. The membership of the SLG includes the Chief Investigator (CI) and an Associate Investigator of the intervention project, who brokered access to the group.

### Recruitment

At the start of the evaluation project, off-the-record interviews were conducted by KML with organizational gatekeepers (ie, MHS managers who were also investigators on the project) to gain a basic understanding of strategic decision making in the MHS. In addition, the Executive Director invited the evaluator (KML) to brief participants on the project (October 2013) and informally observe a senior leadership meeting (November 2013).

The SLG emailing list was then used to invite participants to workshops and interviews; this ensured that data were collected only from active decision makers and members of the SLG. All participants were contacted at least 3 times for each data collection point, unless they had previously withdrawn from the study. All communication regarding meeting scheduling was logged, including cancellations and rescheduling. Signed consent was obtained from all participants during their first in-person contact with the study. The project was approved by the Human Research Ethics Committee of the partner MHS, with approval being valid from December 5, 2013 to January 9, 2019.

#### Adaptations to Recruitment

Owing to instability in the membership and meeting schedule of the SLG during 2014-15, participant access for interventions and their evaluation became limited. There was also marked organizational staff turnover, with 9 managerial departures, 8 internal promotions, and 4 external hires. Only 6 of the recruited participants remained in the senior management group for the duration of the project.

For the intervention, engagement became reliant on the interests of individual participants, with ad-hoc one-on-one and small group discussions replacing workshops and presentations with the entire SLG. These interactions were facilitated by the dual membership of the CI as both a researcher and participant.

For evaluation, the scope of the project was expanded to include the experiences of the researchers in responding to this environment. Hence, all researchers who were actively involved in the project between 2014 and 2016, defined by attendance at a minimum one project meeting, were invited to participate in interviews in 2017. Furthermore, research team meeting minutes and notes were retrospectively added to the data analysis, with the consent of the research team and the appropriate ethics amendments.

### Intervention

#### Phase 1: Development of a Conceptual Framework

In the first phase, we will analyze the components of a mental health system and develop a generic framework for the simulation model. Subphases will be (1) scenario generation; (2) entity modeling; (3) parameter modeling; (4) temporal changes modeling; and (5) output.

##### Scenario Generation

Participants will be consulted to determine the scenarios to be modeled. However, 3 general model scenarios are planned: (1) policy change affecting the structure of services; (2) population distribution changes; and (3) organizational innovation in the delivery of care models.

##### Entity Modeling

The main entities of this model are patients, staff, services, and resources (eg, budget allocation), with their interactions representing the activities of an actual health care system. A priority-based queuing model [21,22] will be adopted to allocate services based on patient severity and need. A patient will be allocated for a set of services within a selected service component where a particular service is provided by a set of staff members who use a set of resources.

##### Parameter Modeling

Parameter modeling consists of 2 components, namely, calculation and prediction. During the model building phase, this module will calculate arrival and transition rates and the length of stay using the observational data for a given scenario. During the validation and predictive assessment phases, the values of the above parameters will be predicted taking into consideration the expected changes and the data for validation.

##### Temporal Changes Modeling

The temporal changes that mainly influence the mental health system are demographics and technological changes. Demographic changes largely result from changes in birth and migration rates and will be projected from data available through the Australian Bureau of Statistics.

##### Output

For assessing the impact of a service component or policy option in terms of health gain, we plan to use 2 quantitative measures: quality-adjusted life year (QALY) and disability-adjusted life year (DALY).

QALY is an outcome measure for evaluating the burden of disease. It takes into account both the quantity and the quality of the extra life provided by a health care intervention or policy option and is calculated as the product of the life expectancy and the quality of the remaining years. While QALY is useful for cost-effectiveness analysis, weights used in calculation are not linked to a particular disease, condition, or disability, but are rather based on an individual’s health state.

DALY is a measure of disease burden that captures both morbidity and mortality effects for a wide range of disorders and interventions and the baseline information for the health status in Australia is readily available [23]. The DALY incorporates disability weight that assigns different weights at different ages; and disability weight values for particular mental health disorders and different categories (eg, mild and severe) are available in the literature.

The model will allow end users to choose either of the measures through a graphical user interface. Apart from QALY and DALY, impacts on blocking rate and resource utilization will be investigated, and specific illness outcomes could be considered, depending on the focus of the scenario chosen.

#### Phase 2: Integration With Arena

A specialist in modeling will build a simulation model in Arena [24], a widely used discrete-event simulation tool. It will include different modules that represent process, entity, queue, and others elements. The output of the simulation model will be used to create custom statistics, a built-in feature in Arena. Once developed, it will require minimal effort by MHS managers to upload instances of a particular entity or update them as required, offering flexibility and the capacity for managers to use the system autonomously.

#### Phase 3: Validation of the Model

The data collected from the MHS will be divided into 2 sets. One will be used for model building, while the other will be used for validation, the 2 sets being mutually exclusive. To test quantitatively how adequately the model represents the actual system, within the service components of a particular scenario, we will compare the model output with actual historical (ground truth) values. For this, the values of model output parameters (eg, changes in QALYs, waiting time, and resource utilization) will be compared with their respective ground truth values through a statistical goodness-of-fit test (eg, chi-square test). Similarly, to test the model for predictive performance, the output of the model in response to the validation data will be quantitatively compared with their corresponding ground truth values (known because the validation set is also part of the available historical data). Strong agreement between the model output and the corresponding actual values will assure the model’s accuracy in emulating the actual system.

#### Phase 4: Implementation of the Model

The project will also involve the provision of training to MHS managers in the use of the simulation modeling tool to guide decision making regarding the configuration and resourcing of MHS. Such training and the availability of the simulation model will enable MHS managers to adopt new approaches to service management, with their decision making being underpinned by much stronger evidence than is currently available.

#### Adaptations to the Intervention

To capitalize on participant interest stimulated by the October 2013 project briefing, a program logic modeling (PLM) workshop was scheduled during the SLG meeting in December 2013, with a follow-up workshop scheduled for July 2014. The aim was to generate inputs for the creation of the simulation models (phase 1 of the project) and to continue participant engagement in the project. The PLM workshops were facilitated by an experienced external contractor. In the first workshop, participants were prompted to identify strategic issues challenging the MHS and their consequences for the organization, staff, and consumer. The second workshop aimed to validate the outputs of the previous workshop and confirm the organizational structure of the MHS prior to integration with the modeling software.

### Evaluation

#### Process Change

Research on problem structuring methods and group model building claim that the process is often more influential than the final model in the decision making of users [25,26]. The development of PLM as a significant element of the project allowed this claim to be tested.

Immediate changes in decision-making process will be evaluated through the observation of participants’ interactions during simulation workshops and a pilot self-report survey on workshop effectiveness. Survey questionnaire items were derived from a frequency analysis of the claimed benefits of PLM in journal papers [27-31], focusing on the PLM methodology and evaluation. The literature search yielded a list of 39 nonunique descriptors. The content analysis of these descriptors revealed 4 overarching categories—clarity, communication, action, and buy-in. Items were selected for face validity and based on the prevalence of categories in the literature. Hence, clarity (6 items) and communication (4 items) were more heavily represented than action and buy-in (2 items each). This yielded 14 items rated on a Likert scale (5=*strongly agree* and 1=*strongly disagree*; Multimedia Appendix 1).

#### Mental Model Change

The primary outcome of interest is a change in the strategic decision making of the SLG to incorporate greater amounts of evidence. This will be captured by comparing the decision-making mental models of SLG members pre- and postintervention, within the group (similarity), and to an ideal standard (ie, rational decision making and accuracy). Mental model similarity and accuracy are both predictive of increased group performance [32,33].

To extract mental models of current decision-making, participants were asked, “If a new staff member arrived today, what would you tell them about how decisions get made by the management team?” They will then be prompted with statements such as “and before that?” or “after that?” Concept maps of current decision-making processes were created during the interview and validated against interview transcripts. To assess the test-retest reliability of the elicitation method, during the exit interview, participants were again asked, “If a new staff member arrived today, what would you tell them about how decisions get made by the management team?.”

#### Adaptations to Evaluation

Adaptations to the intervention necessitated an adaptation to the evaluation. Of most impact was the lack of group meetings or workshops, meaning that group processes were no longer able to be directly studied through observation or questionnaire. The *ad-hoc* nature of meetings with participants exacerbates the lack of structured data collection, necessitating a greater reliance on the document analysis and interview content in the analysis stages.

The document analysis includes business plans, strategic documents, meeting minutes, and other documentation relevant to the decisions addressed by the study. Documents were released by the MHS Office of the Executive Director and the CI. These documents were used to establish a decision-making context and track the development of decisions prior to the initiation of this project. Furthermore, public document sources that provide participant demographics information, organizational information, and government policy information were accessed when required.

Interview content was also expanded to include more open-ended reflections from participants and researchers on the project, discussing topics of expectations, learning, and possible external factors affecting the implementation (Tables 1 and 2).

**Table 1:** Semistructured interview questions for researchers.

Topic	Example questions
Background	Firstly, can you share any reflections on the project in general?
Project evaluation	What were your original plans and expectations for the project? How well do you think the reality met your expectations?
	How do you think the organisational change at the MHS^a^ affected the project?
	What has this project achieved?
	Do you believe that we have affected change at the MHS? How? Why?
	Finally, if you could describe the project in one word, what would it be?
Lessons learned	What were the strengths and weaknesses of our approach? What would you change for next time?
	What have you, personally, learnt/gained from this project?
	Has this project changed the way you understand: … mental health? …modelling? …strategic decision making? …research projects?
	If you could provide one piece of advice for another group doing similar work, what would it be?

^a^MHS: mental health service.

**Table 2:** Semistructured interview questions for managers.

Topic	Example questions
Background	So, we last talked about this time in 2014, two years ago [remind them of the timeline]. So I just wanted to get your thoughts and feelings on the last two years in the mental health service (MHS)?
Organizational change	You predicted [insert prediction] about the period of change in the MHS. To what extent has your expectation been met?
	What are your predictions for the future of the SLG^a^?
Mental models	And how about now? Do you have a sense of a decision-making process for the MHS? What is that? Were there any intermediate models?
	Who makes strategic decisions for the MHS at the moment?
Evaluation of current SLG performance	If you could describe your feelings about the SLG in one word, what would it be?
Simulation project evaluation	I also wanted to get a sense of how the modelling project sat within all of this organisational change. How relevant was the modelling project to you as a member of the SLG?
	What were your expectations for the project [refer to 2014 interview transcripts]? Were they met?
	Has your personal decision-making practice changed? How? Why?
	If you could describe your feelings about the simulation project in one word, what would it be?

^a^SLG: senior leadership group.

### Data Collection and Management

All evaluation data collection was conducted by KML to maintain the separation between the researchers conducting the intervention and the evaluation of the intervention.

A total of 18 interviews were audiorecorded and transcribed verbatim, with 1 participant refusing a recording of the exit interview, instead of allowing note-taking. All audiorecordings, notes, and documentation were imported into the qualitative data analysis software NVivo 10 for analysis [34].

Field notes were kept by KML documenting the time, date, general content, and personal emotions and thoughts associated with contact with participants.

To maintain a close relationship to the data and participants, study data are stored in an identifiable format in password-protected files and folders on password-protected computers located at the core administration site. These can only be accessed by the research staff. The study data will be stored for a minimum of 7 years, after which these may be confidentially destroyed.

### Analyses

All evaluation analyses will be conducted by KML, with an external senior qualitative researcher providing guidance and analysis checks where required.

#### Mental Models

The content analysis of the interview transcript was used to review and refine the interview diagram into a concept map. Each individual’s content map was transcribed into a matrix formation with an arbitrary distance of 1, and input into the network analysis software JPathfinder [33,35] for quantitative analysis. Participants’ individual models were compared with each other in a pairwise fashion, generating a matrix of similarity values (Pathfinder r). This range was used to represent the overall group model similarity.

Group-level concept maps will be created manually by combining all current concept maps, noting agreement by the count of participants who mentioned each concept or a similar construct. This procedure will be repeated for the second time-point. Group-level mental models at each time-point will then be compared against each other to assess any changes over the intervention period.

#### Linguistic Coding Framework

Linguistic coding will be used to assess the process effects of the PLM workshops. Initial codes were derived from the literature on the benefits of problem structuring methods and group model building [36-38] and then matched to concept descriptions and behavioral examples (Table 3). Transcripts of the group discussions will be assessed for similarity to ideal behavior as defined by the literature, for example, equal participation among participants [38].

**Table 3:** Behavioral coding examples for PLM workshops.

Coding variable	Behavioral or linguistic cue
Problem exploration	“But we don’t know…” “We need to know…”
Discussion of alternatives	“What about…” “Or we could…”
Participation	Pattern of speaking duration by gender, role, and over the course of the workshop
Voice	Interjections Speaker participation relative to seniority
Information sharing	“In our service…” “From my point of view…”
Clarification of meaning	“What do you mean?” “I mean that…” “Do you agree?”
Agreement	“I agree” “Yes”
Disagreement	“No” “I don’t agree”

#### Thematic Analysis

Participant and researcher interviews will be analyzed using thematic analysis. Open coding will be used to explore the data prior to an iterative process of thematic refinement involving member checks and the exploration of alternative interpretations. These interpretations will be presented to participants, providing them with the opportunity to provide further comment. Furthermore, the researcher-participants will be involved in the written publication of the analysis, ensuring shared ownership of the project evaluation and recommendations.

## Results

The project was funded in 2012 and recruitment was completed in October 2016. Sixteen managers participated in at least one data collection (see Table 4 for a summary of participation patterns). Three researchers participated in interviews with the evaluator (KML), with another 2 providing written responses to question prompts.

**Table 4:** Sample participation patterns across data collection points to date.

Participant	Workshop 1 (N=8)	Interview 1 (N=9)	Workshop 2 (N=8)	Interview 2 (N=9)
1	✓	✓	✓	✓
2	✓	✓	✓	✓
3	✓	✓	✓	✓
4	✓		✓	✓
5		✓	✓	✓
6	✓	✓		
7		✓	✓	
8		✓	✓	
9		✓		✓
10	✓			✓
11	✓			
12	✓			
13		✓		
14			✓	
15				✓
16				✓

Primary data collection has been completed. Data analysis is currently under way, with parallel member checking ongoing. The first results are expected to be submitted for publication in late 2018.

## Discussion

This research protocol outlines the implementation and evaluation of simulation modeling in the planning of MHS in Australia. As a case study, this research design has both advantages and limitations. The iterative design of the intervention allows easy adaptation to the changing organizational context; however, this comes at the cost of clear data points for quantitative evaluation. This is addressed by favoring a qualitative case study approach for evaluation, at the cost of generalizable findings. However, given the lack of reporting on simulation implementation in the past, such deep access and analysis provide a unique opportunity to understand the realities of translational research in this area.

While the methods used allow for feedback from senior staff, which includes direct-care staff and a consumer representative, the organizational level of the modeling intervention does not readily allow for the incorporation of other direct feedback from consumers, family members, or nonmanagerial staff. However, following the completion of the project, we expect that the modeling system will be a valuable decision support tool to be used by MHS managers, which will be integrated into the process of decision making around service configuration and allocation of resources within the MHS. This provides the potential for future follow-up studies measuring the intervention impact for patients, families, and nonmanagerial staff.

The challenges faced by the project thus far, especially the instability of the health care context, are not unusual. Hence, lessons from this research have the potential to improve the implementation of future research projects, providing greater evidence-based service planning for the mental health sector in Australia.

## Appendix

Multimedia Appendix 1 Workshop evaluation questionnaire.

Multimedia Appendix 2 ARC Grant Assessor Reports.

## Abbreviations

CI: chief investigator
DALY: disability-adjusted life year
MHS: mental health service
PLM: program logic modeling
QALY: quality-adjusted life year
SLG: senior leadership group
