# Supplementary material for: Simulation Modeling for Psychiatric Service Planning: Protocol for a Mixed-Methods Study
Source: JMIR Res Protoc. 2018 Nov 23;7(11):e11119. doi: 10.2196/11119 (PMC6301811; doi:10.2196/11119)
Supplement: Multimedia Appendix 1 [file resprot_v7i11e11119_app1.pdf]

# Workshop evaluation questionnaire

**Please indicate to what extent you agree or disagree with the following statements?**

| This workshop… | Strongly agree | Agree | Neither agree nor disagree | Disagree | Strongly disagree |
|---|---|---|---|---|---|
| …engaged me in the decision-making process | ☐ | ☐ | ☐ | ☐ | ☐ |
| …helped me think clearly about goals & outcomes | ☐ | ☐ | ☐ | ☐ | ☐ |
| … fostered a common language | ☐ | ☐ | ☐ | ☐ | ☐ |
| …fostered discussion | ☐ | ☐ | ☐ | ☐ | ☐ |
| …structured knowledge about the program | ☐ | ☐ | ☐ | ☐ | ☐ |
| …made assumptions explicit | ☐ | ☐ | ☐ | ☐ | ☐ |
| …got everyone 'on the same page' | ☐ | ☐ | ☐ | ☐ | ☐ |
| … documented the program | ☐ | ☐ | ☐ | ☐ | ☐ |
| …promoted communication | ☐ | ☐ | ☐ | ☐ | ☐ |
| …identified gaps in program logic | ☐ | ☐ | ☐ | ☐ | ☐ |
| …highlighted key performance indicators | ☐ | ☐ | ☐ | ☐ | ☐ |
| …helped me look at the whole picture | ☐ | ☐ | ☐ | ☐ | ☐ |
| …clarified intended outcomes | ☐ | ☐ | ☐ | ☐ | ☐ |
| …improved my understanding of the program | ☐ | ☐ | ☐ | ☐ | ☐ |
