# Supplementary material for: Simulation Modeling for Psychiatric Service Planning: Protocol for a Mixed-Methods Study
Source: JMIR Res Protoc. 2018 Nov 23;7(11):e11119. doi: 10.2196/11119 (PMC6301811; doi:10.2196/11119)
Supplement: Multimedia Appendix 2 [file resprot_v7i11e11119_app2.pdf]

LP110200061 assessors reports

Assessor A

Investigator(s): Investigators' track records are persuasive. The mix of disciplinary backgrounds is broad. I would have preferred others with qualitative backgrounds to complement the team - see comments below.

Significance and Innovation: Modelling of mental health services is an important initiative. Simulation is indeed an accepted and productive means to providing insight into the resilience of existing systems, resources and practices. The application of simulation in mental health planning may be innovative - this is not my field of expertise. I would expect any simulation program to be informed not just by quantitative modelling but also by qualitative assessment - that is, assessment that engages with people who are experiencing those services: patients, families of patients, clinicians, and so forth. More on this below.

Approach and Training: As presented in the proposal, the approach is mostly quantitative. At one point, post-implementation interviews and focus groups are mentioned, to be conducted by the PhD student. Then, on page 14 the PhD student is stated to be conducting interviews on quite a different (and potentially more continuous) basis. The proposal is therefore not clear on whether the simulation model design will occur with input from those whom the model seeks to benefit, managers, and perhaps patients. The proposal is also not strong reflecting contemporary design thinking, which has moved on from mono-disciplinary approaches and 'designers' designs' to favouring multi-disciplinary approaches, and user design participation over and above user feedback. Training: the proposal incorporates one PhD stipend. The PhD project appears to include, besides conceptualisation and implementation of the model (huge tasks), a more qualitative study of design needs and outcomes. The CIs are stated to be in charge of training the PhD student. Given CIs backgrounds, providing training with respect to conceptualisation and implementation of the model is justified. Training with regard to qualitative skills does not appear to be catered for.

National Benefit: A strong example showing modelling service development and aiding in service planning might be of national benefit.

Partner Organisation Commitment: Partner organisation commitment and involvement is persuasive.

Improvements (if not applicable, please enter n/a): This proposal would benefit from making two changes. First, the proposal needs expanding of its qualitative components. These are currently buried, slightly inconsistent and inadequately worked out. This might involve invitation to a qualitative researcher to join the team, ensure the design process appropriately takes account of social complexities affecting existing services and approaches, and perceived impacts of particular approaches to service planning. Second, the proposed model might well serve health service managers, but to what extent would it serve patients and families? How are patients and families going to be involved in this work? These are questions that need to be addressed and answers need to be justified.

Comments (if not applicable, please enter n/a): n/a

Assessor B

Investigator(s): The investigators have relatively good track records.

Significance and Innovation: The proposal addresses an important problem. The application of simulation techniques in the target domain is innovative and can deliver significant value. The proposal is well-written.

Approach and Training: The research plan as well as the PhD training plan have been appropriately formulated.

National Benefit: There is a clear case for national benefit.

Partner Organisation Commitment: The PO has made an appropriate commitment.

Improvements (if not applicable, please enter n/a): n/a

Comments (if not applicable, please enter n/a): n/a
